# Swimming and *Campylobacter* Infections^1^

**DOI:** 10.3201/eid1008.030924

**Published:** 2004-08

**Authors:** Daniela Schönberg-Norio, Johanna Takkinen, Marja-Liisa Hänninen, Marja-Leena Katila, Suvi-Sirkku Kaukoranta, Leena Mattila, Hilpi Rautelin

**Affiliations:** * University of Helsinki, Helsinki, Finland;; † Helsinki University Central Hospital, Helsinki, Finland;; ‡ Kuopio University Hospital, Kuopio, Finland;; § North Karelia Central Hospital, Joensuu, Finland; 1 This study was in part presented at the 12th International Workshop on *Campylobacter*, *Helicobacter* and Related Organisms, Aarhus, Denmark, September 6–10, 2003.

**Keywords:** Campylobacter, Campylobacter jejuni, Campylobacter coli, Gastroenteritis, Case-control study, epidemiology, Finland, dispatch

## Abstract

A matched case-control study was conducted to study risk factors for domestically acquired sporadic *Campylobacter* infections in Finland. Swimming in natural sources of water was a novel risk factor. Eating undercooked meat and drinking dug-well water were also independent risk factors for *Campylobacter* infection.

*Campylobacter jejuni* and *C. coli* are leading causes of human bacterial gastroenteritis in industrialized countries (1,2). In 1998, in Finland, the number of reported *Campylobacter* cases exceeded that of salmonella for the first time (2). A similar increase in *Campylobacter* incidence is evident in other industrialized countries (1,3), but the reason for this finding remains unknown (1).

Most human C*ampylobacter* infections are sporadic, and a seasonal peak in the distribution of the infections occurs during the summer months in several countries, including Finland (1–4). A variety of risk factors for *Campylobacter* infections have been identified, including handling and eating poultry (1,3,5–7) and drinking unpasteurized milk (1,3,5,7) or untreated water (1,8,9). In Finland, several waterborne outbreaks have been reported (10), but risks associated with sporadic *Campylobacter* infections are largely unknown (Table 1).

**Table 1:** Matched multivariate analysis of significant risk factors for domestically acquired sporadic *Campylobacter* infection, July–September 2002, Finland^a^

Risk factor	Adjusted OR	95% CI	2-tailed p
Tasting or eating raw or undercooked meat	10.79	1.31–89.09	0.0272
Drinking water from a dug well	3.36	1.37–8.24	0.0082
Swimming in water from natural sources	2.80	1.23–6.39	0.0145

^a^OR, odds ratio; CI, confidence interval.

In our case-control study, we identified risk factors for and possible sources of infection for domestically acquired sporadic *Campylobacter* infections in Finnish patients from three geographic areas during the seasonal peak from July 1 to September 30, 2002.

## The Study

Three clinical microbiology laboratories that served patients in the southern, central, and eastern parts of Finland participated in this multicenter, matched case–control study. A case-patient was defined as a person with stool culture, collected during the study period and tested at one of the three laboratories, that was positive for *C. jejuni* or *C. coli*. Patients from both outpatient clinics and hospitals were included. When a *Campylobacter*-positive patient was identified, personnel from the microbiology laboratory contacted the clinic or hospital for more information on the patient's recent travel history. If the patient had not traveled abroad within 2 weeks before illness, that patient's physician was contacted by phone and was asked to send to the patient information about our study and a questionnaire and a prepaid envelope to be returned to the researchers.

Two age-, sex-, and municipality-matched controls were chosen for each case-patient. Controls were selected from the Population Register Center, Espoo, Finland, an official register of all Finnish residents. Potential controls were contacted by mail and asked to fill in a questionnaire and mail it back in a prepaid envelope. Exclusion criteria for the controls were *Campylobacter* infection, at least 3 loose stools per day, abdominal pain, or fever 30 days before filling out the questionnaire. If the questionnaire was not returned within 2 weeks, a new pair of controls was chosen, leading to a maximum of four controls per case.

The questionnaire sent to patients included questions on the disease, travel in and outside of Finland, dietary intake of food items (meat, fish, vegetables, fruit, and dairy products), quality of drinking water, contact with pets and other domestic animals, and swimming in water from natural sources. The controls answered similar questions except for those concerning illness. Case-patients and controls were excluded if they had traveled abroad within 2 weeks before illness (case-patients) or filling in the questionnaire (controls). The study was approved by the Ethics Committee of the Hospital District of Helsinki and Uusimaa.

For sample-size calculation, the case-control ratio was 1:2. The exposure level among patients and controls was assumed to be 30% and 15%, respectively. The study was based on the estimate that 97 patients would be needed for the 5% significance level with 80% power. Only patients with at least one matched control were accepted for the final study set. Data entry was performed by EpiData 2.1b (EpiData Association, Odense, Denmark), and statistical analyses were made with EpiInfo 2002 (Centers for Disease Control and Prevention, Atlanta, GA). For risk factors with 95% confidence interval (CI) above one, conditional logistic regression examined these independently related to *Campylobacter* infection.

Of the 316 patients with stool culture–verified *Campylobacter* during the study period, 208 had no known foreign travel; the 634 controls also had not traveled outside of Finland. A total of 151 (73%) patients and 309 (49%) controls returned the questionnaire. Of the patients, 11 were excluded because of traveling abroad (according to the questionnaire), 3 for misunderstanding or missing information, 5 for having too long a delay (>37 days) between onset of symptoms and answering the questionnaire, and 11 because the delay between symptoms and answering the questionnaire could not be defined. In addition, a matched control was unavailable for 21 patients. Of the controls, 172 were excluded for the following reasons: traveling abroad (17 controls), gastrointestinal symptoms (56 controls), missing information (21 controls), and previous *Campylobacter* infection (2 controls); 76 were omitted because of the lack of a matching case. The final analysis was made up of 100 patients and 137 controls.

A total of 99 patients were infected with *C. jejuni* and 1 with *C. coli*. All cases were sporadic and not associated with any known outbreaks. Regional distribution and demographic characteristics of patients and controls are presented in Table 2. Patients and controls were matched in doubles (66), triples (31), and quadruples (3). Patients filled in the questionnaires within 3 to 37 days from onset of illness, with a median delay of 16 days. The median interval between onset of illness of the patients and their controls responding to the questionnaire was 32 days. The median delay between patients and controls filling in the questionnaire was 15 days. The total number of exposures analyzed was 82. Factors significantly associated with an increased or a reduced risk for *Campylobacter* infection are shown in Table 3.

**Table 2:** Patient characteristics

Characteristic	Patients, N = 100 (%)	Controls, N = 137 (%)
Sex		
Male	42 (42)	56 (41)
Female	58 (58)	81 (59)
Age (y)		
1–4	4 (4)	6 (4)
5–9	1 (1)	2 (2)
10–19	3 (3)	5 (4)
20–29	13 (13)	18 (13)
30–39	9 (9)	13 (10)
40–49	15 (15)	18 (13)
50–59	26 (26)	32 (23)
>60	29 (29)	43 (31)
Median age (y)	51	51
Municipality		
Helsinki	35 (35)	47 (34)
Kuopio	44 (44)	62 (45)
Joensuu	21 (21)	28 (20)

**Table 3:** Matched univariate analysis of exposure factors for domestically acquired sporadic *Campylobacter* infection, July–September 2002, Finland^a^

Risk factor	Patients, n = 100	Controls, n = 137	Adjusted OR	95% CI	2-tailed p
Increased risk					
Tasting or eating undercooked or raw meat^b^	14/88	3/124	12.00	1.54–93.77	0.0052^c^
Drinking water from a dug well	31/96	22/137	3.19	1.58–6.45	0.0017
Swimming in water from natural sources	48/100	40/134	2.27	1.24–4.16	0.0089
Eating strawberries	70/89	79/124	2.90	1.21–6.95	0.0287
Reduced risk					
Consumption of					
Black and red currants	17/73	73/126	0.17	0.07–0.41	< 0.0001
Blueberries	20/73	56/118	0.43	0.21–0.89	0.0115
Carrots	43/83	89/126	0.44	0.24–0.82	0.0039
Yogurt	51/90	83/121	0.35	0.15–0.85	0.0332
Pasteurized milk	55/93	99/131	0.44	0.22–0.85	0.0075
Cooked or fried fish	50/93	98/128	0.35	0.18–0.67	0.0004
Liver (beef)	4/69	15/105	0.18	0.04–0.87	0.0083
Drinking water produced by a large water plant	52/97	88/137	0.52	0.26–1.02	0.0371
Eating at a friend's house	24/49	44/71	0.35	0.13–0.96	0.0195
Others					
Eating					
Minced meat (pork)	45/83	70/116	0.54	0.28–1.06	0.0438
Minced meat (beef)	64/90	97/128	0.78	0.42–1.46	0.3459
Drinking					
Water produced by a small water plant	23/97	34/137	0.80	0.37–1.72	0.4528
Water from bedrock well	20/97	17/137	1.96	0.89–4.34	0.1210
Bottled water	15/97	27/137	0.75	0.37–1.51	0.3062
Contact with cat	27/88	40/118	0.87	0.43–1.76	0.5768
Contact with dog	53/93	76/128	1.02	0.55–1.89	0.9385
Contact with farm animals	4/83	9/118	0.36	0.06–2.14	0.1426
Eating outside the home	69/98	100/137	0.78	0.41–1.48	0.3686
Eating chicken prepared from					
Nonmarinated pieces	9/71	20/111	0.32	0.10–1.06	0.0274
Marinated pieces	34/81	47/111	0.76	0.38–1.58	0.3613
Nonmarinated strips	11/70	13/114	1.06	0.33–3.46	0.8357
Marinated strips	19/77	40/118	0.61	0.29–1.28	0.1324
Drinking unpasteurized milk	7/80	9/111	1.40	0.45–4.37	0.7768

^a^OR, odds ratio; CI, confidence interval. ^b^Of 14 exposed patients, 13 specified meat type: 8 (57 %) had tasted undercooked poultry, and 5 (36%) had tasted minced meat. ^c^Fisher exact test.

Of the 14 patients who ate undercooked or raw meat, 57% had eaten poultry and 36% minced meat, supporting previous studies that have identified eating undercooked poultry as a risk factor (7,8,11). Except for tasting or eating undercooked chicken meat, preparing or eating chicken was not associated with an increased risk for *Campylobacter* infection in our study.

Of the four significant risk factors in the initial univariate analysis, three were independently associated with *Campylobacter* infection in multivariate analysis: tasting or eating undercooked or raw meat, drinking untreated dug well water, and swimming in natural sources of water (Table 1). At least one of these three epidemiologically associated risk factors was found in 67% of the patients.

## Conclusions

We identified, to our knowledge for the first time, swimming in natural sources of water to be an independently associated risk factor for sporadic *Campylobacter* infection. As the infective dose for *Campylobacter* infection is likely low, contaminated surface water may cause infection through swimming; campylobacters are commonly found in natural waters, such as rivers, streams, and lakes (12). However, in contrast to our study, in a recent Norwegian study (9), swimming in the sea, lakes, and swimming pools was associated with a reduced risk for *Campylobacter* infection.

Our study showed that private water supplies present a significant risk factor for sporadic *Campylobacter* infection. Kapperud et al. (9) also found that exposure to surface water or drinking nondisinfected water caused an increased risk. In Finland, in addition to the 310,000 households that use private wells, approximately 300,000 summer cottages have private water supplies (13). Dug wells are susceptible to surface water contamination. Furthermore, the summer of 2002 was exceptionally dry in Finland, resulting in poor water quality in these wells because of low groundwater levels. In our study, drinking water from a large water plant protected against sporadic *Campylobacter* infection. Large water plants usually have surface water as their source and use multistage purification and disinfection procedures before drinking water is distributed to consumers, which substantially reduces risk for waterborne infections.

Eating strawberries, although a significant risk factor in univariate analysis, was not an independent risk factor in the multivariate analysis. During the same time period but outside the study region, a small cluster of cases was reported for which the suspected source was eating strawberries directly from the field (14).

Reduced risk for the disease was associated with eating other berries, such as red and black currants and blueberries, and carrots. These findings are consistent with the literature (7–9), although no one fully understands the role of these protective factors.

In Finland, because most sporadic *Campylobacter* infections occur during July to September, our study could not identify risk factors that may have varied seasonally. The median age of our patients and controls was considerably high (51 years of age), which may have influenced the results. This age group, however, may be typical for Finland, since in our previous study sporadic, domestically acquired *Campylobacter* infections were frequent in certain parts of the country in elderly men (15).

In addition to the known risk factor of eating raw or undercooked meat, this study clearly identified water as an important risk for domestically acquired *Campylobacter* infections in the summertime in Finland. The novel finding that swimming in water from natural sources was associated with increased risk for infection further emphasizes the importance of other water-related exposure factors.
